# Meta-analysis of classical swine fever prevalence in pigs in India: A 5-year study

**DOI:** 10.14202/vetworld.2018.297-303

**Published:** 2018-03-13

**Authors:** S. S. Patil, K. P. Suresh, S. Saha, A. Prajapati, D. Hemadri, P. Roy

**Affiliations:** Indian Council of Agricultural Research - National Institute of Veterinary Epidemiology and Disease Informatics (ICAR-NIVEDI), PBNO-6450, Yelahanka, Bengaluru, Karnataka, India

**Keywords:** Classical swine fever, India, meta-analysis, pigs, prevalence

## Abstract

**Aim:**

The aim of the study was to determine the overall prevalence of classical swine fever (CSF) in pigs in India, through a systematic review and meta-analysis of published data.

**Materials and Methods:**

Consortium for e-Resources in Agriculture, India, Google Scholar, PubMed, annual reports of All India Coordinated Research Project on Animal Disease Monitoring and Surveillance, and All India Animal Disease database of NIVEDI (NADRES) were used for searching and retrieval of CSF prevalence data (seroprevalence, virus antigen, and virus nucleic acid detection) in India using a search strategy combining keywords and related database-specific subject terms from January 2011 to December 2015 in English only.

**Results:**

A total of 22 data reports containing 6,158 samples size from 18 states of India were used for the quantitative synthesis, and overall 37% (95% confidence interval [CI]=0.24, 0.51) CSF prevalence in India was estimated. The data were classified into 4 different geographical zones of the country: 20% (95% CI=0.05, 0.55), 31% (95% CI=0.18, 0.47), 55% (95% CI=0.32, 0.76), and 34% (95% CI=0.14, 0.62). CSF prevalence was estimated in northern, eastern, western, and southern regions, respectively.

**Conclusion:**

This study indicates that overall prevalence of CSF in India is much lower than individual published reports.

## Introduction

Classical swine fever (CSF) is one of the most important, highly contagious, and fatal viral diseases of domestic pigs characterized by high fever, anorexia, gastrointestinal symptoms, general weakness, conjunctivitis, hemorrhage, cyanotic skin, and leukopenia leading to heavy mortality and substantial economic losses to pig industry [1,2]. The causative agent is CSF virus (CSFV), a small, enveloped RNA virus that belongs to the genus *Pestivirus* within the family *Flaviviridae* [3]. Severity and mortality depend on the virulence of the virus, host and environmental factors, ages, and breeds of pigs [1,4]. The disease is widely prevalent in pig population of Europe, Asia, and South America [5], and a high prevalence of CSFV antibodies in pigs suggests that the disease is endemic in India [5,6].

In India, the first suspected case of CSF occurred in Aligarh in 1944 [7]. Thereafter, subsequently disease was reported in other parts of the country [5]. A total of 611 outbreaks of CSF in India were reported during 2000-2015 [8], which led to heavy economic losses to pig farmers directly through mortality and reproductive losses in affected pigs and indirectly by bringing restrictions on exports of pork and pork products. According to an economic study, India incurs losses of 9.085 million INR each year due to CSF outbreaks in pigs [9].

Meta-analysis is a statistically powerful framework for estimating the magnitude, consistency, and homogeneity of the effect of interest across studies [10]. Recent past years, the prevalence of CSFV antibodies/antigen/nucleic acid was reported by different researchers which gives only isolated status of CSF in different states of India [5,6]. However, the epidemiology of CSF has not been studied systematically, and therefore, the prevalence status of the disease is largely unknown at the country level.

The present study aims to systematically review the existing literature and provides a standard estimate of the prevalence of CSF regionally and country wise. This would pave the way for epidemiological modeling which would help to formulate and evaluate control strategies in the long run.

## Materials and Methods

### Ethical approval

Not Applicable as there were no animal experiments carried out in this study.

### Search strategy

A literature search for publications on the prevalence of CSF studies in India or in different states from January 2011 to December 2015 was performed using the three English databases, namely, Consortium for e-Resources in Agriculture (ICAR-CeRA), Google Scholar, and PubMed. Search was made using the term “CSF”, “CSF and India” and CSF seroprevalence or CSF prevalence or CSF virus infection in India’ in the CeRA, Google search, and PubMed for searching the databases. The criteria for prevalence study in India and it’s various states were defined as follows: (i) Screening of sera for CSF IgG antibody and whole blood for CSFV antigen carried out using the CSFV antibody and antigen enzyme-linked immunosorbent assay (ELISA) kits, respectively, and virus-specific reverse transcriptase-polymerase chain reaction (RT-PCR) [5,11-15]; (ii) Mean prevalence of CSFV antigen in blood samples recorded in different districts of respective states [5,16-20]; and (iii) Annual reports of All India Coordinated Research Project on Animal Disease Monitoring and Surveillance (AICRP on ADMAS) on seroprevalence studies of serum samples collected for detection of CSFV-specific antibody by ELISA kits [8,21]. All reference lists from relevant studies were read to locate additional studies but did not contact any authors of original studies for additional information. Full-text articles were downloaded or obtained through library resources.

### Included criteria

**Figure-1:**
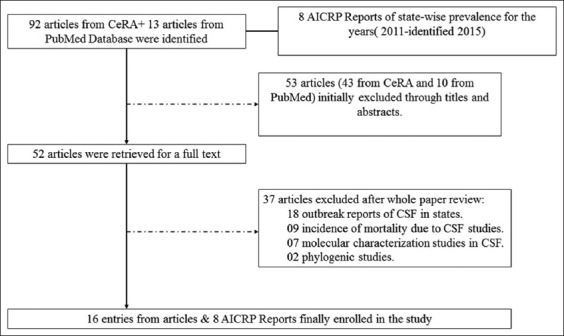
The flow diagram of paper review process (one article may contain 1/more studies).

All the search results were limited to observational studies conducted on both clinically affected and apparently healthy animals. Prevalence study mainly was based on representative pig serum samples and tissue samples collected from different groups of pigs using antibodies or antigen-based ELISA and RT-PCR as a surveillance tool [22-24]. A total of 92 records published in journals were from the CeRA and PubMed databases. Removal of duplicates and initial screening through titles and abstracts yielded 53 papers that were reviewed. After a thorough review, 37 articles were excluded as it accounted for outbreak reports of CSF, molecular characterization, phylogenic studies, and prevalence study of diagnostic potentials. The flow diagram of the review process was shown in Figure-1. Final sample was a total of 9 research articles and 4 annual reports of AICRP on ADMAS. The studies had been based on 17 states of India, and finally, 24 studies were included in the study (Table-1) [6,11,12,15-17,19,21,25,26].

**Table-1:** Characteristics and data summaries of the publications included in the study.

References	States	Study period	Total samples tested	Total positive samples
Nandi *et al*.[6]	West Bengal, Meghalaya, and Nagaland	2011	11	10
	Madhya Pradesh and Maharashtra	2011	171	126
	Rajasthan	2011	46	40
	Andhra Pradesh, Karnataka, and Kerala	2011	296	157
	Jammu and Kashmir, Punjab, and Uttar Pradesh	2011	70	43
Annual Report of AICRP on ADMAS[21]	NER/Manipur	2011	25	3
	Assam	2011	100	18
	Manipur	2011	100	12
	Meghalaya	2011	100	43
	Andhra Pradesh	2011	844	81
	Madhya Pradesh (M.P)	2011	100	34
Rout *et al.*[25]	Uttar Pradesh (U.P)	2012	1120	86
George *et al.*, [26]	Assam	2012	48	8
Deori *et al*.[19]	Assam	2012	98	57
Annual Report of AICRP on ADMAS [21]	Maharashtra	2012	58	51
Shivaraj *et al*.[12]	Karnataka	2013	517	173
Ahuja *et al*.[16]	Meghalaya	2014	264	138
	Manipur	2014	252	97
Malmarugan *et al*.[11]	Tamil Nadu	2014	110	90
Annual Report of AICRP on ADMAS [21]	Andhra Pradesh	2014	65	5
Choori *et al*.[17]	Karnataka	2015	218	89
Rajbongshi *et al*., [15]	NER	2015	325	45
Annual Report of AICRP on ADMAS [21]	Uttar Pradesh	2015	1120	54
	Jammu & Kashmir	2015	100	38

NER=North-Eastern Region, AICRP=All India Coordinated Research Project, ADMAS=Animal Disease Monitoring and Surveillance

### Quality of the studies

Risk of bias among the included studies was evaluated using a quality assessment checklist. Simple score system (2 for “yes,” 0 for “no,” or 1 for “unsure”) based on the following question was used [27].

1. Whether the result is corresponding to the objective mentioned?
2. Whether the sampling strategy was described in details?
3. Whether the period of the study clearly mentioned?
4. Whether the test used in the research was meeting the included criteria?

### Data extraction

The characteristics of each included study were extracted onto pre-designed Excel forms. The following data were extracted from the selected studies: The author, publication year, state, district, and number of samples tested with results [28]. In the process of data extraction, all the data obtained were validated for further analysis.

### Strategy for data synthesis

A meta-analysis of the prevalence of CSF in pig populations in India was conducted using the software Review Manager 5.2 [29]. A total of 24 studies (Table-1) were included from different states of the country. Since seroprevalence of CSF was reported from 18 states of the 29 states and 7 union territories, further classification into 4 different zones was carried out to estimate the heterogeneity between studies. The states which reported the seroprevalence of CSF were categorized into four zones as mentioned as follows:

**Table-2:** Details of heterogeneity (%) and prevalence (95% CI) studies of CSFV among pig populations at all India and zone-wise level.

Regions	Total studies	Total sample size	*I*^2^ value	τ^2^ value	p value	Random effects			Fixed effects		
	
						Prev.	Lower	Upper	Prev.	Lower	Upper
All India	24	6158	98%	2.069	<0.01	0.37	0.24	0.51	0.30	0.28	0.31
North	4	2410	99%	2.622	<0.01	0.20	0.05	0.55	0.11	0.10	0.13
East	10	1323	95%	1.088	<0.01	0.31	0.18	0.47	0.35	0.33	0.38
West	4	375	95%	1.437	<0.01	0.74	0.45	0.90	0.65	0.59	0.70
South	6	2050	98%	2.117	<0.01	0.34	0.14	0.62	0.33	0.30	0.35

Prev*=Prevalence, CSFV = Classical swine fever virus

Northern zone - Jammu and Kashmir, Punjab, and Uttar Pradesh.

Eastern zone - Assam, Manipur, Meghalaya, Mizoram, Nagaland, Tripura, and West Bengal.

Western zone - Rajasthan, Maharashtra, and Madhya Pradesh.

Southern zone - Undivided Andhra Pradesh, Karnataka, Kerala, and Tamil Nadu.

The Chi-square test was conducted to assess the heterogeneity. It was evaluated using Tau-squared (τ^2^) value and its level of significance [30]. Results on meta-analysis for random effect model were used if the heterogeneity between studies was found to be significant and higher τ^2^ [8]. I^2^ statistic which is used to describe the percentage of variation between studies was used to indicate the degrees of heterogeneity between studies. If the value of I^2^ is <50%, we use a fixed effect model to calculate the point estimate of seroprevalence and its 95% confidence interval (CI) [29]. As summary data were used, and therefore, no ethical approval was needed in this present study.

## Results

**Figure-2:**
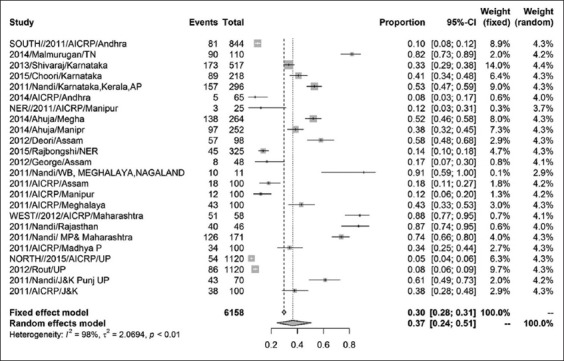
Forest plot of all Indian classical swine fever prevalence.

**Figure-3:**
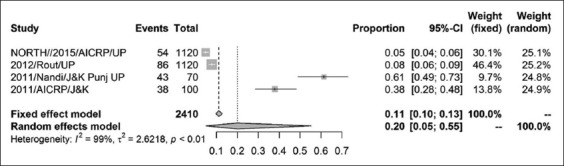
Forest plot of east zone classical swine fever prevalence.

**Figure-4:**
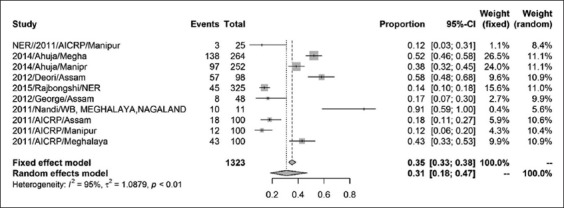
Forest plot of west zone classical swine fever prevalence.

**Figure-5:**
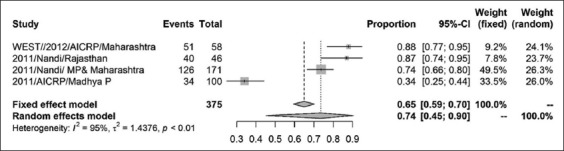
Forest plot of north zone classical swine fever prevalence.

**Figure-6:**
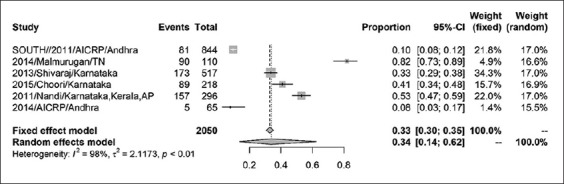
Forest plot of south zone classical swine fever prevalence.

The prevalence of CSFV in India was found to be 37% (95% CI=0.24, 0.51) for a sample size of 6158 as shown by the forest plot (Figure-2). Stratification into different zones leads to the estimation of prevalence of north zone as 20% (95% CI=0.05, 0.55) with a sample size of 2410 (Figure-3) and east zone as 31% (95% CI=0.18, 0.47) having a sample size of 1323 (Figure-4), whereas, the prevalence of west zone was estimated to be as high as 74% (95% CI=0.45, 0.90) with a sample size of only 375 (Figure-5). The prevalence of south zone was estimated to be 34% (95% CI=0.14, 0.62) having a sample size of 2050 (Figure-6).

## Discussion

The present study was intended to know the overall prevalence of CSFV in India by meta-analyses of reports on the prevalence of CSF. Many Indian researchers are working at their level to explore epidemiology of CSF in pig population, but available data are fragmented and not showing the situation at the country level. Large data set is important for projecting country level CSF prevalence and to identify the severely affected regions and mobilization of resources. Meta-analysis has become the standard for quantitative evidence synthesis offering a broadly accepted and statistically powerful framework for integrating and adding value to previously published large databases containing raw or partially annotated information. The review searched 3 major bibliographic databases through the internet using a search strategy. In addition, data mentioned in annual reports of AICRP on ADMAS [21], Bengaluru, also were taken into account for the calculation of total prevalence.

Both methods, namely, antibody and antigen detection in the sample used in the different studies were included for analysis. Antigen or antibody ELISA was most commonly employed in most studies to know the prevalence of CSF because of convenience in processing a large number of samples and higher sensitivity and specificity. Some workers used RT-PCR for the screening of samples for the detection of virus presence which is more sensitive than that of ELISA. These diagnostic methods were compared for their routine screening of CSF infections and were shown to have good compliance with each other [31], so the testing method was unlikely to be a significant source of heterogeneity in this analysis. Quality scores for the included study ranged from 5 to 8 which indicate relevancy of article with the study.

A systematic review of tools to assess the quality of observational studies examining incidence or prevalence concluded that no consensus exists as to which individual criteria should be assessed to establish methodological quality. This methodology increases the statistical power of the results by enlarging the number of analyzed reports. The present study indicates that the prevalence of CSF is strongly associated with the sample size and estimates that the prevalence of CSFV in India to be 37% (95% CI=0.24, 0.51) for a sample size of 6,158 as shown by the forest plot. This finding is much lower than the previous pan India surveillance finding of 594 serum samples from 12 states and 287 tissue samples tested from 13 states of India using commercial ELISA kits in which mean prevalence of CSFV antibodies in suspected sera was 63.3% (376/594), and CSFV antigen in the suspected samples was 76.7% (220/287) [6]. Higher seroprevalence may be due to the screening of samples from suspected animals while present analysis includes both healthy and suspected animals.

Region-wise analyses of CSF prevalence revealed the high prevalence of CSF (74%) in the western region. The reasons for high prevalence may be attributed to skewed distribution of pig population, non-random sampling, and risk-based sampling that is justified by low τ^2^ value of 1.437 when compared with overall τ^2^ value of 2.069. The synthesized prevalence rates of CSFV in eastern and southern regions were 31% and 34%, respectively, which is nearer to the overall prevalence rate of 37% with moderate heterogeneity measured by τ^2^ and adoption of appropriate sampling methodology. It is to mention that the pig population in eastern and southern regions is normally distributed [32]. In the north region, the prevalence rate was 20% which was lower compared to the overall prevalence rate. The weak surveillance system may be the reason of low prevalence in the north region. It was observed that there was no substantial publication bias in our findings that reflects true prevalence of CSFV as is evident from the heterogeneity index measured (Table-2). However, higher prevalence rates in the west region may be attributable to the sampling bias not to the publication bias. The year-wise distribution of reports on the prevalence of CSFV used in this study is stated to be uniform, and hence, time-specific bias is likely to negligible.

### Limitations

Limitations in our meta-analyses included studies which were obtained from the reports other than the reports of AICRP on ADMAS, which highly depends on the passive mode of surveillance. Poor sampling methodology and sampling from high endemicity areas do likely to contribute to overestimation of prevalence. The potential bias in the estimation of prevalence could be due to variation among the diagnostic tests, non-separation of reports of active and passive surveillance, and limited reporting of information.

## Conclusion

Meta-analysis of CSF prevalence in pigs was found to be 37%. This study indicated that overall prevalence of CSF in pigs in India is much lower than individual published reports. Higher seroprevalence of CSF in pigs in earlier reports may be due to screening of samples from CSF suspected pigs while present analysis included both healthy and suspected animals.

## Authors’ Contributions

SSP conceptualized the aim of the study, designed, planned, and supervised the analysis and corrected the manuscript. KPS performed all analysis, prepared the graphs, figures and tables. SS and AP drafted the manuscript. DH and PR provided conceptual support, and critically reviewed the manuscript. All authors read and approved the final manuscript.
